# Preliminary Anatomical and Imaging Characterization of Vascular and Neural Changes in Dogs with Perineal Hernia

**DOI:** 10.3390/vetsci13040353

**Published:** 2026-04-03

**Authors:** Mercedes Marañón-Almendros, Luis Avedillo, Gonzalo Sánchez-Banderas, Nieves Martín-Alguacil

**Affiliations:** Research Group GIMCAD 971005-UCM, Departmental Section of Anatomy and Embryology, School of Veterinary Medicine, Universidad Complutense de Madrid, 28040 Madrid, Spain; mdemaran@ucm.es (M.M.-A.); luiavedi@ucm.es (L.A.); gosanc07@ucm.es (G.S.-B.)

**Keywords:** caudal rectal artery, external anal sphincter, fluoroscopy, internal iliac artery, perineal arteries, perineal hernia, perineal nerves, vascular variations

## Abstract

A perineal hernia in dogs results from a failure of the pelvic diaphragm and is often accompanied by a displacement of the pelvic and abdominal organs. However, detailed descriptions of accompanying vascular and neural alterations are limited. This study uses a combination of fluoroscopic imaging and anatomical dissection to characterize the perineal arterial and neural anatomy in two control dogs and three dogs with unilateral or bilateral perineal hernias; however, only one control underwent fluoroscopy, and neural dissection was performed on one control and on the bilateral case. In the control specimen, the vascular pattern followed the classical NAV arrangement. In contrast, the hernia cases exhibited marked variability, including altered origins and lengths of the dorsal and ventral perineal arteries; long- or short-type internal iliac and internal pudendal arteries; and displacement or deterioration of the superficial perineal nerve. These changes were most pronounced in the dog with a bilateral hernia, which was accompanied by severe degeneration of the levator ani and coccygeus muscles and herniation of the colon, urinary bladder, and urethra. Fluoroscopic findings closely corresponded with anatomical observations, demonstrating the value of imaging in the preoperative assessment of vascular variations. Overall, this study provides a comprehensive description of the neurovascular and muscular modifications associated with perineal hernias and emphasizes the need for individualized anatomical evaluations to optimize surgical planning and reduce intraoperative risks.

## Simple Summary

A perineal hernia is a condition that primarily affects older intact male dogs. It occurs when the muscles that support the pelvic organs weaken or fail. As these muscles weaken, organs such as the rectum, colon, or bladder may move into the perineal region. This creates visible swelling and causes difficulty defecating or urinating. Although this condition is well known in veterinary practice, the exact changes that occur in the area’s blood vessels and nerves are not fully understood. This study examined the perineal regions of two healthy dogs and three dogs with perineal hernias; however, only one control underwent contrast enhanced fluoroscopy, and neural dissection was performed on one control and on the dog with bilateral hernia. We found that dogs with hernias showed significant differences in the position and structure of the arteries and nerves compared to healthy dogs. The changes were more severe in dogs with long-standing or bilateral hernias where the organs had moved into the hernial sac, severely damaging the supporting muscles. Understanding how anatomy changes in these cases can help veterinarians plan safer, more effective surgeries. Our findings underscore the importance of considering individual anatomical variations when treating perineal hernias in dogs.

## 1. Introduction

A precise understanding of the vascular anatomy of the pelvic cavity is essential for interpreting normal structures and pathological conditions. Any organ, muscle, or soft tissue in this region may be supplied—or compromised—by variations in the internal iliac artery (IIA) and its branches [1,2,3]. One clinical situation in which these variations are particularly relevant is perineal hernia (PH), a disorder in which the pelvic diaphragm weakens, allowing tissues to displace into the perineal region [4,5,6]. Despite the importance of the internal iliac and perineal arteries (PAs) for surgical planning and tissue viability, most available information comes from anatomical dissections [7,8,9]. Little is known about how these vessels appear on diagnostic imaging.

Although Avedillo et al. [2,3] have provided valuable, large-scale descriptions of pelvic vascular anatomy in dogs, the broader veterinary literature remains limited in its detailed documentation, particularly regarding clinically relevant variations in the internal iliac artery and its perineal and rectal branches. While classical descriptions of the internal pudendal artery (IPA) and its branches—the caudal rectal artery, dorsal perineal artery (PA), and ventral PA—are widely accepted [10,11,12,13,14,15,16], several authors have reported alternative branching patterns that may have significant clinical implications [2,3]. These variations are especially relevant in pathological conditions that distort the perineal region, such as PHs, because displacement of pelvic structures can alter the normal location of vessels and nerves.

PH is a clinically significant condition in dogs. It predominantly affects older males, with reports indicating that up to 96% of affected animals are male, with only a small minority being female [4,17,18,19]. The disorder typically develops in middle-aged to geriatric dogs, with an average age of 7–9 years in males and slightly older in females [4]. Certain breeds, such as Pinschers, Poodles and Maltese, appear to be predisposed. PH is frequently associated with comorbidities that contribute to its development and complicate its management [4]. A PH in dogs occurs when the muscles that typically support the tissues around the anus weaken. This allows pelvic or abdominal structures to shift into an abnormal position [4]. Surgical correction is the preferred treatment, typically involving internal obturator muscle transposition [20,,21,22,23,24,25]. However, cases with severe muscle atrophy or recurrence may require reinforcement with polypropylene mesh or alternative reconstructive techniques [26,27,28,29,30], such as semitendinosus muscle flap transposition. Among the potential complication after perineal herniorrhaphy fecal incontinence occurred in 10% of the dogs [5] and damage to the innervation of the external anal sphincter may be the cause. Although this condition is widely recognized in clinical practice, its underlying anatomical and neurovascular features are not well documented. In particular, the PAs and nerves that supply the external anal sphincter have received limited attention despite their direct relevance to surgical repair and postoperative continence. Injury to the perineal nerves, particularly the caudal rectal nerve and the branches of the superficial perineal nerve, may result in reduced anal sphincter tone, altered perineal sensation, or fecal incontinence. Understanding the local neural anatomy is important to minimize the risk of iatrogenic nerve injury. Despite the clinical importance of these anatomical relationships, few studies have combined imaging techniques with traditional dissection to characterize the vascular anatomy of the perineal region under normal and pathological conditions [31,32,33]. Variations in the arterial supply may influence tissue viability, complicate surgical dissection, or increase the risk of intraoperative bleeding. Inadvertent injury to key nerves can result in serious functional consequences [1,2]. Most descriptions of the perineal vasculature in dogs are based on standard anatomical patterns, and reports of atypical configurations are scarce. Similarly, although the caudal rectal, superficial perineal, and deep perineal nerves are known to contribute to sphincter innervation, their precise location in dogs with PHs has not been systematically evaluated.

Anatomical variations in the PAs and nerves can significantly impact the surgical treatment of these conditions. The traditional anatomical model, which features a shorter dorsal PA that only vascularizes the dorsal part of the perineum, is present in 45.7% of cases [2]. However, the perineal nerve does not follow this artery; instead, it continues alone to innervate the external anal sphincter and the skin [1]. This variation may require careful dissection to avoid nerve damage during surgery. The long type of the dorsal PA, found in 12.5% of cases, is longer and vascularizes both the dorsal and ventral parts of the perineum [2]. It anastomoses with the smaller ventral PA. The perineal nerve always follows this long artery [1]. The perineal trunk is present in 41% of cases and involves a common trunk from which the dorsal and ventral PAs emerge [2]. The perineal nerve follows the perineal trunk’s route [1]. Understanding these anatomical variations is essential for veterinarians to effectively treat and manage these conditions and improve the quality of life for affected dogs. Comprehensive knowledge of these pathologies and their surgical solutions ensures that veterinarians can provide optimal care for their patients.

Although contrast-enhanced CT is a powerful tool for vascular imaging, our study required a technique that could be applied to cadaveric specimens while avoiding vascular overlap. Fluoroscopy allows us to visualize contrast-filled arteries in cadavers in real time and directly correlate radiographic vascular patterns with anatomical dissections. Although fluoroscopy is currently limited to cadaveric models, it provides a practical method for obtaining clear, non-superimposed images by injecting contrast into a single internal iliac artery. In this context, fluoroscopy is a valuable tool for understanding perineal vascular anatomy and may inform the development of future preoperative angiographic techniques. When combined with detailed anatomical dissections, fluoroscopic images can reveal vascular patterns, confirm anatomical variations, and demonstrate how these structures might appear in future diagnostic imaging. This study uses paired fluoroscopic images and latex-injected dissections to document the vascular anatomy of the internal iliac and PAs in control dogs and dogs with PHs. Correlating imaging findings with direct anatomical evidence highlights the potential of diagnostic imaging to identify clinically relevant vascular variations that are currently undetectable in live patients. These observations lay the groundwork for future advancements in perineal vascular imaging and enhance our understanding of the underlying anatomy of PHs.

In addition to vascular anatomy, the innervation of the external anal sphincter and surrounding perineal structures is essential for continence and postoperative function [34]. However, the relationship between PHs and alterations in perineal innervation has received limited attention. This study uses a combined approach of fluoroscopy and anatomical dissection to characterize vascular variations in the IIA and its perineal and rectal branches, as well as to describe innervation of the external anal sphincter. We examined two control dogs and three clinical cases of PHs to compare normal and altered anatomy. This integrated morphological analysis provides new insights into the vascular and neural organization of the canine perineal region and offers practical information for improving surgical strategies in PH repair.

## 2. Materials and Methods

### 2.1. Animals and Study Design

This study evaluated a total of five dogs, including two controls and three clinical cases. This study only included male dogs because perineal hernias predominantly occur in males, and no female specimens with this condition were available during the study period. While this reflects the known epidemiology of pelvic hernias, the absence of female samples limits our ability to assess potential anatomical variations involving ovarian, uterine, or other sex-specific structures. Table 1 summarizes the main characteristics of dogs included in the study.

#### 2.1.1. Vascular Study (Fluoroscopy and/or Arterial Dissection)

This study included three dogs. One control dog was examined through fluoroscopic vascular imaging, followed by arterial dissection. One dog with left PH (Case 1) was examined through fluoroscopic vascular imaging and arterial dissection. And one dog with a unilateral PH was examined through arterial dissection to assess vascularization.

#### 2.1.2. Vascular and Neural Dissection Study

The second study included two dogs. One was a control dog, and the other was a dog (Case 3) with a bilateral PH that underwent dissection of its vascular and neural structures.

#### 2.1.3. Specimen Acquisition

All cadavers were obtained from dogs euthanized by their veterinarians due to advanced age, chronic comorbidities, or severe clinical conditions. One control dog was euthanized due to severe chronic renal failure associated with leishmaniasis. All specimens were obtained immediately after euthanasia and prepared for anatomical study in accordance with European Union regulations (Directive 2010/63/EEC) and Spanish legislation (RD 53/2013).

### 2.2. Fluoroscopic Imaging

Fluoroscopy was only used to examine the left side in both the control dog and the case of a left-sided hernia. To prevent misinterpretation due to overlap of vessels from both hemipelves, contrast medium was injected exclusively into the left IIA. Urografin^®^ (Bayer Hispania S.L., Sant Joan Despí, Barcelona, Spain), a 370 mg/mL injectable and perfusion contrast medium containing 0.1 g sodium amidotrizoate and 0.66 g meglumine amidotrizoate per milliliter, was used for vascular opacification. This corresponds to 370 mg of iodine per milliliter. We administered the contrast agent by directly injecting it into the iliac segment of the aorta. This procedure was performed dynamically and simultaneously with fluoroscopic acquisition. We adjusted both the injection volume and pressure in real time while monitoring the progressive filling of the arteries of interest. Only the minimum pressure necessary for adequate vascular opacification was applied to ensure that no anatomical distortion or displacement occurred during the procedure. A continuous video was recorded during contrast injection to monitor the progressive filling of the vessels (see Videos S1 and S2 in the Supplementary Materials). The images used for analysis were selected at the optimal filling phase, before the contrast diffused into the surrounding tissues. Fluoroscopic imaging was performed using a Philips BV25 Gold C-arm system (Philips Medical Systems, Amsterdam, The Netherlands). Lateral projections were obtained to document the course, branching pattern, and relative position of the IIA, caudal gluteal artery, IPA, and their perineal and rectal branches. Real-time fluoroscopic images were acquired to visualize the arterial branching pattern within the pelvic and perineal regions. Fluoroscopic imaging was performed with a field of view of 22 cm × 22 cm and an approximate pixel size of 0.18 mm. The X-ray energy ranged from 65 to 75 kVp and the tube current from 1.2 to 1.6 mA. These values were automatically adjusted by the system according to tissue density and contrast opacification. Images were acquired in pulsed mode at 7.5 frames per second (fps), which provided sufficient temporal resolution for real-time monitoring of vascular filling while avoiding unnecessary radiation exposure. The imaging and dissection were carried out in different facilities within the Faculty of Veterinary Medicine at the University of Complutense of Madrid. Fluoroscopy was performed in the diagnostic imaging suite of the Veterinary Teaching Hospital and subsequent dissections were conducted in the Department of Anatomy and Embryology’s dissection room. Specimens were transported between locations without manipulation that could alter the anatomical arrangement of the structures under study.

### 2.3. Vascular Injection Technique

The pelvic vasculature was injected with colored latex in all cases to allow for detailed visualization of arterial branching patterns. The IIA was cannulated proximally and filled with latex under controlled pressure until the entire pelvic arterial tree was filled. The filling of the pelvic arterial tree was confirmed by directly observing the superficial vessels turn red when latex was introduced. Minor differences between the fluoroscopic and dissection images likely reflect the sequential injection of the contrast medium, followed by the introduction of latex. However, these variations were minimal and did not alter the course or disposition of the vessels. Specimens were allowed to be cured 24 h before imaging or dissection.

### 2.4. Anatomical Dissection

The pelvis and both hind limbs were fixed together as a single anatomical block. Only this region was preserved. The fixation process did not alter the anatomical location of the structures, which allowed for accurate dissection and analysis. All specimens were prepared using conventional anatomical methods and injected with colored natural latex to enhance visualization of the vascular system. Látex Natural Concentrado ALTAIR^®^ (Altair S.A., Lambare, Paraguay) was used for vascular perfusion. It had an initial solid content of 60%. We prepared an aqueous dilution to achieve a final concentration of 40–45%, which provided the appropriate viscosity for the homogeneous filling of the arterial tree without causing obstruction or extravasation. The mixture was colored with 3–5% SODISE^®^ Red Latex Dye (Bretagne, France) by volume to ensure optimal visibility during anatomical dissection. Both components were mixed immediately before perfusion to maintain stability and prevent premature thickening. Latex injection was performed manually by applying constant, controlled pressure based on prior experience with this technique. The latex injection continued until the superficial skin capillaries were visibly filled, ensuring adequate, homogeneous vascular opacification without overdistension. Both the left and right internal iliac arteries and their major branches were then exposed, photographed, and sketched. Standard approaches to the pelvic cavity, ischiorectal fossa, and perineal region were used to identify the IIA, pudendal artery, dorsal and ventral PAs, and their branches. Throughout the dissection process, particular attention was given to the origin and variations in the PAs, as well as the course and position of the superficial perineal nerve.

### 2.5. Assessment of Neurovascular Structures

The neurovascular assessment involved a detailed examination of the course and distribution of the caudal rectal, superficial perineal, and deep perineal nerves. Attention was given to their relationship with the external anal sphincter and adjacent vascular structures. During dissection, the perineal nerve was carefully exposed and traced to document its topography in relation to the dorsal PA. This process also allowed us to identify the presence of a perineal trunk and determine whether it is presented as a long-type or short-type configuration. All neural structures were photographed, drawn, and described in situ to accurately depict their anatomical relationships and capture variations associated with PHs.

### 2.6. Assessment of Hernia Anatomy

For dogs with PHs, the anatomical assessment focused on the location, extent, and contents of the hernia sac, which is defined as a peritoneal or retroperitoneal outpouching that protrudes through a weakened or altered pelvic diaphragm. The anatomical assessment focuses on the location, extent, and contents of the hernia sac, as well as the condition of the pelvic diaphragm muscles. In the unilateral case, clinical examination and anatomical dissection confirmed that the hernia was right-sided, showing displacement of structures only on the right side while the left perineal region remained intact. The assessment also focused on the condition of the pelvic diaphragm muscles. The hernia sac was located between the coccygeus and levator ani muscles in all affected hemipelves, with the latter showing varying degrees of structural deterioration. The herniated contents included the urinary bladder, portions of the urethra, and, in some cases, segments of the colon. Attention was given to the displacement and distortion of vascular structures in relation to the hernia sac and to the course of the superficial perineal nerve. The relative position of the vessels and nerves, whether lateral or medial to the hernia sac, was documented through direct observation and photographic records. This provided a detailed understanding of how a PH alters the topography of the perineal region. Information regarding the chronicity of the perineal hernia was not available, as the specimen was received post-mortem without clinical history; therefore, the duration of the condition prior to anatomical examination could not be determined.

### 2.7. Histological Study

As a preliminary study, a histological evaluation of the pelvic diaphragm muscles was performed on the control 2 dog, as well as on the unilateral and bilateral hernia cases (Cases 2 and 3). Samples of the levator ani and coccygeus muscles were collected immediately after dissection to ensure comparable anatomical regions across individuals. The tissue specimens were fixed in 10% neutral buffered formalin for 48 h, dehydrated through a series of ethanol solutions, cleared in xylene, and embedded in paraffin. Serial transverse and longitudinal sections (4–5 µm thick) were prepared using a Microm HM 325 rotary microtome (MICROM International GmbH, Walldorf, Germany) and stained with hematoxylin and eosin. The slides were examined under a Leica DM 500 light microscope (Wetzlar, Germany) at 4×, 10×, and 40× magnification. This approach enabled a thorough evaluation of muscle fiber architecture, nuclear morphology, and connective tissue organization in control samples as well as in samples collected from the coccygeus and levator ani muscles. All slides were evaluated under a light microscope at multiple magnifications to compare the healthy musculature of the control dog with the structural alterations observed in the unilateral and bilateral hernia cases.

### 2.8. Data Recording and Classification

The data collection focused on documenting arterial branching patterns and, when applicable, perineal innervation in all five specimens. For each dog, observations were recorded during fluoroscopy, if performed, and through detailed anatomical dissection of the latex-injected vasculature. The course, origin, and relationships of the IIA and its major branches, including the caudal gluteal, internal pudendal, caudal rectal, dorsal perineal, ventral perineal, and urethral arteries, were examined systematically on both sides of the pelvis. Additional attention was given to the position of vessels relative to the hernia sac and the condition of the pelvic diaphragm muscles in dogs with PH. When neural structures were included in the study, the superficial perineal nerve and its branches were carefully exposed and traced toward the external anal sphincter. All findings were documented through written notes, photographs, and anatomical drawings to ensure the accurate and consistent recording of vascular and neural topography.

## 3. Results

### 3.1. Vascular Study (Fluoroscopy and/or Arterial Dissection)

**Figure 1:**
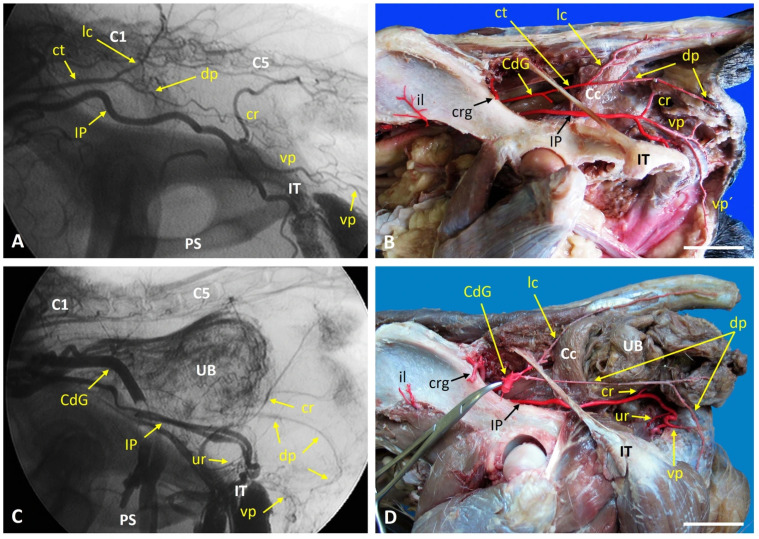
Fluoroscopic and anatomical images of the left hemipelvis in a control dog and Case 1, showing normal and abnormal perineal vascular patterns. Paired fluoroscopic images and corresponding anatomical dissections of the left hemipelvis in a control Cocker Spaniel and Case 1. In the control dog (**A**,**B**), contrast injection at the origin of the left internal iliac artery outlines the expected NAV-type branching pattern, as confirmed by anatomical dissection. One anatomical variation was observed: a common trunk (ct) giving rise to the lateral caudal and dorsal PAs. In Case 1 (**C**,**D**), fluoroscopy and dissection reveal a markedly elongated dorsal perineal artery (dp) that forms an anastomosis with a reduced ventral PA. C1: First caudal vertebra; C5: Fifth caudal vertebra; Cc: Coccygeal muscle; CdG: Caudal gluteal artery; cr: Caudal rectal artery; crg: Cranial gluteal artery; ct: Common trunk; dp: Dorsal perineal artery; il: Iliolumbar artery; IP: Internal pudendal artery; IT: Ischial tuberosity; lc: Lateral caudal artery; PS: Pelvic symphysis; UB: Urinary bladder; ur: Urethral artery; vp: Ventral perineal artery; vp′: Dorsal scrotal branch of the ventral perineal artery. Scale bar: 2 cm.

In the control dog, fluoroscopy of the left internal iliac artery and its branches revealed the normal, expected vascular pattern (Figure 1A). This pattern was confirmed during anatomical dissection (Figure 1B). The internal iliac artery gave rise to the umbilical, internal pudendal, and caudal gluteal arteries. The internal pudendal artery branched into the prostatic, ventral perineal, and penile arteries. The caudal gluteal artery gave rise to the iliolumbar, cranial gluteal, lateral caudal, and dorsal PAs. The only variation observed in the control specimen was a common trunk that gave rise to both the lateral caudal and dorsal PAs. In case 1 (left-sided perineal hernia), fluoroscopy and dissection revealed a markedly elongated dorsal PA that extended caudally and formed an anastomosis with a reduced ventral PA (Figure 1C,D). This configuration differed from the normal pattern seen in the control dog and was located adjacent to the herniated region.

**Figure 2:**
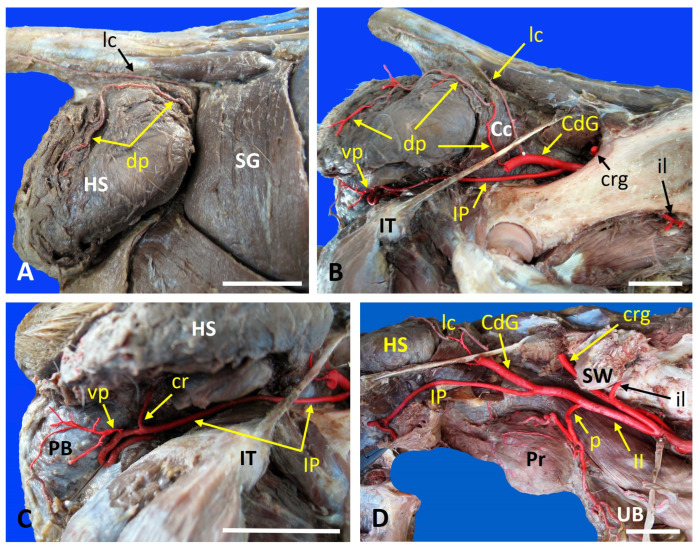
Anatomical Dissection Planes of Case 2. Dog with a right-sided perineal hernia that was injected with colored latex to visualize the vascular anatomy. The images illustrate the altered arrangement of vessels within the herniated region, shown consecutive planes from superficial to deep: (**A**) The superficial dissection plane shows the hernial sac and the course of the dorsal perineal artery. (**B**) An intermediate dissection plane, with the superficial gluteal muscle withdrawn to reveal the course of the internal pudendal artery, as well as the dorsal and ventral perineal arteries. (**C**) A closer magnification of the internal pudendal artery and the caudal, rectal and ventral perineal arteries. (**D**) A deep dissection plane showing the complete vascular branching pattern of the internal iliac artery after removal of the coxal bone. Cc: Coccygeal muscle; cdg: Caudal gluteal artery; cr: Caudal rectal artery; crg: Cranial gluteal artery; dp: Dorsal perineal artery; HS: hernia sac; II: Internal iliac artery; il: Iliolumbar artery; IP: Internal pudendal artery; IT: Ischial tuberosity; lc: Lateral caudal artery; p: Prostatic artery; PB: Penis bulb; Pr: Prostate; P: Penile bulb; SG: Superficial gluteal muscle; SW: Sacral wing; UB: Urinary bladder; vp: Ventral perineal artery. Scale bar: 2 cm.

In Case 2 (right-sided perineal hernia), both the dorsal and ventral PAs were present; however, the ventral PA did not give rise to a caudal rectal branch (Figure 2). The internal iliac artery was unusually long, while the internal pudendal artery was shorter than normal. These features represent a modified vascular arrangement associated with the herniated tissues. Although vascular variations were observed in both the control and hernia cases, they fell within the normal anatomical spectrum and did not affect group classification, which was based on the presence or absence of a hernia.

### 3.2. Vascular and Neural Dissection Study

**Figure 3:**
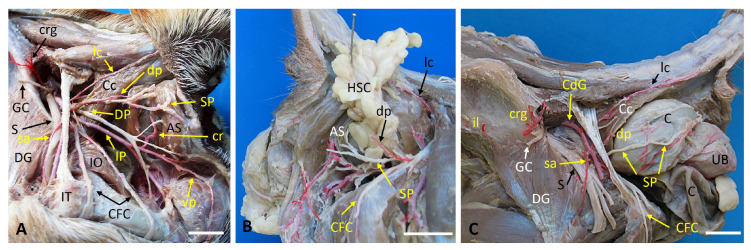
Lateral anatomical dissections of a control dog and Case 3 illustrating the perineal nerve distribution and its symmetry in a dog with bilateral perineal hernia. (**A**) The left side of the control dog. Vascularization and innervation of the ischiatic fossa after removal of the gluteal musculature. (**B**) Right side of the dog with perineal hernia. The contents of the hernial sac are visible, exposing the vascular and nerve structures. (**C**) Left side of the dog with a hernia. After the gluteal musculature has been removed, the vascularization and innervation of the ischiatic fossa can be observed, including the modifications caused by the presence of the hernial sac. AS: external anal sphincter muscle; C: colon; Cc: coccygeal muscle; cdg, caudal gluteal artery; CFC: caudal femoral cutaneous nerve; cr: caudal rectal artery; crg: cranial gluteal artery; DG: deep gluteal muscle; DP deep perineal nerve; dp: dorsal perineal artery; HSC, hernia sac content; il, iliolumbar artery; IO: internal obturator muscle; IP: internal pudendal artery; IT: Ischial tuberosity; lc: lateral caudal artery; S: Sciatic nerve; sa: satellite artery; sp: superficial perineal nerve; UB: urinary bladder; vp: ventral perineal artery. Scale bar: 2 cm.

Figure 3 illustrates the vascular and nerve anatomy of the perineal region in a control dog and in case 3, a dog with a bilateral perineal hernia. In the control animal, the internal pudendal artery, vein, and nerve run caudomedially over the internal obturator muscle. The pudendal nerve gave rise to the caudal rectal nerve, which traveled with the caudal rectal artery toward the external anal sphincter. The deep perineal nerve traveled alongside the ventral PA, while the superficial perineal nerve accompanied the dorsal PA to supply the perineal skin and external anal sphincter (Figure 3A). In Case 3, the right-sided hernia (Figure 3B) contained a short dorsal PA, which was consistent with the normal pattern. The hernia sac was positioned between the coccygeus and levator ani muscles with partial structural loss of the levator ani. The superficial perineal nerve passed caudally beyond the dorsal PA and gave off a branch to the external anal sphincter before continuing caudoventrally. Both the dorsal PA and the superficial perineal nerve were medial to the hernia sac. On the left side (Figure 3C), a short dorsal PA was present again. The hernia sac occupied the space between the coccygeus muscle and the markedly compromised levator ani muscle. The colon, urinary bladder, and part of the urethra were displaced into the hernia sac. The superficial perineal nerve passed caudally over the dorsal PA. Very fine, deteriorated nerve fibers extended toward the external anal sphincter; however, their full course could not be confirmed. On this side, the dorsal PA and the superficial perineal nerve were positioned laterally to the hernia sac.

**Figure 4:**
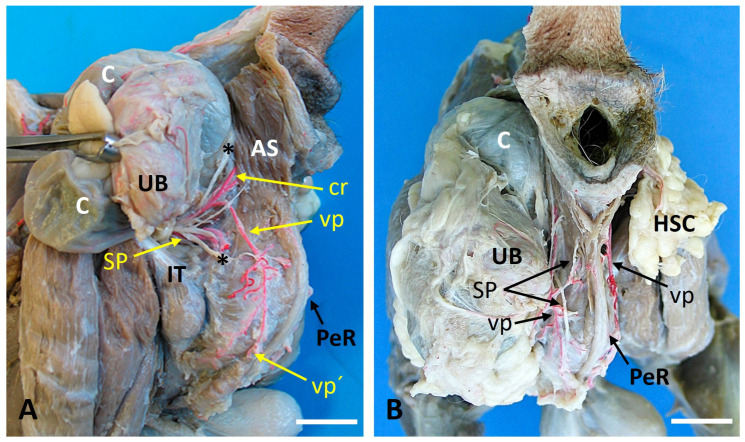
Caudal and caudolateral anatomical views of Case 3 illustrate the vascular and nerve distribution in a dog with a bilateral perineal hernia. (**A**). Caudolateral view of the left ischiorectal fossa of Case 3 is shown, along with the cutaneous nerve branches marked by an asterisk (*). (The asterisk marks the cutaneous branches of the perineal nerve.) (**B**). Caudal view of the perineal region of Case 3. AS: External anal sphincter muscle; C: Colon; cr: Caudal rectal artery; HSC: Hernia sac content; IT: Ischial tuberosity; PeR: Penile retractor muscle; spn: Superficial perineal nerve; UB: Urinary bladder; vp: Ventral perineal artery; vp´: Dorsal scrotal branch of the ventral perineal artery; Scale bar: 2 cm.

Figure 4 shows the bilateral displacement of tissues and altered neurovascular topography in Case 3. The hernia sacs appear as two rounded protrusions that occupy the ischiorectal fossae. On the left side, a long dorsal PA arose from the pudendal artery and traveled caudally to anastomose with a short ventral PA. On the right, the internal iliac artery was unusually long, giving rise to a shortened pudendal artery before branching into the perineal vessels.

### 3.3. Histological Study

**Figure 5:**
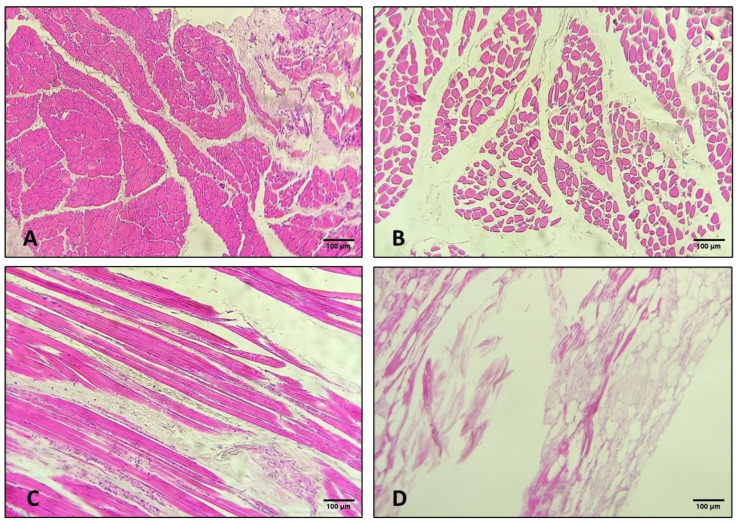
Histological sections (10×) of the left coccygeal and levator ani muscles from a control dog (**A**,**C**) and Case 3 (**B**,**D**) are shown. The sections illustrate normal architecture versus degenerative changes associated with a bilateral perineal hernia.

A histological evaluation revealed significant differences between the control samples and the samples obtained from the affected muscles (Figure 5).

The control specimens displayed normal skeletal muscle architecture with cohesive fibers, prominent nuclei, and well-defined transverse striations (Figure 5A,C). There was no evidence of inflammatory infiltration. Case 3 showed degenerative changes were evident in both the right and left coccygeus muscles, including loss of fiber cohesion, widening of the inter-fascicular spaces, frequent fiber fragmentation, and sparse, small, pyknotic nuclei (Figure 5B). Transverse striations were largely preserved except in a few fibers that showed an amorphous, eosinophilic transformation. Similar, albeit more severe, alterations were observed in the levator ani muscles. The right levator ani sample was scarce and showed markedly separated fibers with multifocal myofiber fragmentation and pyknotic nuclei. The left levator ani muscle exhibited significant architectural disruption, extensive degeneration and necrosis. These changes are consistent with chronic mechanical overload and impaired perfusion associated with long-standing perineal herniation. Much of the muscle tissue had been replaced by adipose tissue (Figure 5D). Overall, the histological findings in the coccygeus and levator ani muscles were consistent with degenerative changes and necrotic changes in the latter.

## 4. Discussion

This study provides a detailed characterization of the neurovascular anatomy of the perineal region in dogs with PHs, integrating fluoroscopic imaging and anatomical dissection to document normal and altered configurations. In control dogs, the vascular pattern followed the classical NAV arrangement, which served as a reliable baseline for comparison [11,15]. In contrast, the three hernia cases exhibited deviations involving the origins of the PAs, altered course of the superficial perineal nerve, and displacement of pelvic and abdominal organs into the hernial sac. These findings highlight the anatomical complexity of PHs and the importance of recognizing individual vascular and neural variations during surgical planning. A key observation was the variability in arterial and neural arrangements in PH dogs, particularly in advanced or bilateral cases. While the NAV pattern was consistent in the control dog, hernia cases showed deviations that appear to correlate with muscular disruption and organ displacement. Recurrent features included altered origins of the dorsal and ventral PAs, long- or short-type internal iliac and internal pudendal arteries, and changes in the trajectory of the superficial perineal nerve. These modifications likely reflect congenital variation and secondary changes induced by chronic herniation, such as stretching, displacement, or compression of neurovascular structures. This variability underscores the need for individualized anatomical assessments during surgery, as unexpected vessel or nerve positions may increase the risk of intraoperative complications.

Previous anatomical studies have reported variability in the dorsal and ventral PAs, whereas the superficial perineal nerve has traditionally been described as a stable structure with predictable relationships to the coccygeus and levator ani muscles [1]. However, clinical reports of PHs frequently describe displacement or distortion of these structures in chronic or bilateral cases [35,36]. The present findings align with these observations, demonstrating that neurovascular anatomy becomes increasingly variable as muscular support deteriorates. The altered course of the superficial perineal nerve and the inconsistent origin or length of the dorsal PA in hernia cases suggest that chronic herniation induces secondary anatomical changes rather than merely exposing preexisting variations, especially in the perineal nerves. This distinction is clinically relevant because surgeons may encounter fragile nerve branches that are not predicted by standard anatomical references.

From a clinical perspective, the anatomical variations documented in PH dogs have important implications for surgical repair. Displacement of the dorsal and ventral PAs and altered course of the superficial perineal nerve increases the risk of inadvertent vascular or neural injury during dissection [37,38,39]. Long- or short-type internal iliac and internal pudendal arteries may modify the expected location of key branches [2,3], and deterioration or caudal displacement of the superficial perineal nerve, may contribute to postoperative complications, such as reduced perineal sensation or impaired external anal sphincter function [37,38,40]. Notably, the superficial perineal nerve did not consistently accompany the dorsal PA, despite the common surgical practice of using the artery as a landmark to preserve innervation. This variability reinforces the need for careful, individualized identification of neurovascular structures rather than reliance on expected anatomical patterns.

Another important finding was the significant muscle degeneration associated with PHs, particularly in the levator ani and coccygeus muscles. While control samples showed normal architecture, affected dogs exhibited fiber separation, fragmentation, nuclear pyknosis, and, in severe cases, adipose replacement. The left levator ani muscle in the bilateral case showed the most advanced degeneration. Although only one control dog was available for histological comparison, degenerative and necrotic changes were consistently localized to the herniated region and displayed a pattern typical of chronic mechanical overload and impaired perfusion. These lesions were present in multiple affected dogs and absent in muscles outside the herniated area, supporting the interpretation that they were secondary to the PH rather than incidental findings. Because the histological findings were not quantitatively assessed across specimens, the observed muscle alterations should be interpreted descriptively and as supportive evidence rather than definitive proof of a uniform myopathic process.

The bilateral involvement and severity of the changes in the levator ani muscle highlight its pivotal role in the pathogenesis of PHs. This deterioration contributes to mechanical failure of the pelvic diaphragm and influences the displacement of neurovascular structures [8,23,41]. As the levator ani loses integrity, the dorsal and ventral PAs and the superficial perineal nerve may shift medially or laterally depending on the direction and extent of herniation. These findings support the hypothesis that PH is a progressive condition driven by muscular degeneration that results in secondary anatomical distortion rather than a purely mechanical defect [8,35,38]. In unilateral cases, anatomical alterations were present but localized to the affected side. In contrast, the bilateral case demonstrated more profound and symmetrical disruption, with muscular collapse, organ displacement, and altered relationships among the perineal vessels and nerves. The presence of pelvic organs such as the colon, urinary bladder, and urethra within the hernial sac illustrates the severity of bilateral involvement and its capacity to distort regional anatomy beyond what is typically observed in unilateral cases. These observations suggest that bilateral hernias represent a more advanced disease stage characterized by chronicity and progressive muscular degeneration.

Organ displacement was a prominent feature of more advanced hernia cases, especially bilateral presentations [42]. The mechanical pressure exerted by herniated organs may stretch or compress the PAs and nerves, potentially accelerating degenerative changes or altering their functional capacity. This displacement contributes to clinical signs commonly associated with PHs, such as dyschezia, tenesmus, and urinary obstruction, and exacerbates distortion of local neurovascular structures [7,8,9,43]. These findings reinforce the concept that PH is a dynamic condition in which progressive muscular failure and organ displacement interact to reshape regional anatomy in clinically significant ways [39,44].

Chronic increases in intra-abdominal pressure, hormonal influences in intact males, and age-related degeneration of the levator ani and coccygeus muscles likely contribute to this process [8,35,38,45]. As the pelvic diaphragm weakens, the hernial sac expands and exerts mechanical traction on adjacent vessels and nerves, explaining the elongated or displaced dorsal PA, the altered course of the superficial perineal nerve, and the variable branching patterns observed.

The combined use of fluoroscopy and anatomical dissection in this study underscores the diagnostic value of imaging in evaluating PHs [31,32,33]. Fluoroscopy was particularly useful for visualizing the IIA and its major branches, allowing identification of long-or short-type arterial patterns and deviations from the NAV arrangement prior to dissection. Although fluoroscopy is not recommended clinically for evaluating perineal vessels, CT-based angiographic techniques offer superior diagnostic value and are the most appropriate method for evaluating perineal vascular anatomy, especially in complex or recurrent cases.

Overall, PHs in dogs are associated with substantial and often unpredictable alterations to the regional vascular and neural anatomy. These changes are driven by progressive degeneration of the pelvic diaphragm and displacement of pelvic and abdominal organs. Fluoroscopy in this study served solely as a pre-dissection visualization tool, providing an overview that contextualized subsequent anatomical findings. These insights have direct implications for surgical planning, emphasizing the need for individualized assessment of neurovascular structures to minimize intraoperative risks and improve outcomes.

The conclusions of this study must be interpreted in light of the limited number of specimens. The dissection series included only five individuals, two of whom served as controls, and one control exhibited an anatomical variation that reduces generalizability. The findings should therefore be understood as descriptive observations rather than definitive population-level characteristics. Future studies with larger sample sizes are essential to confirm the consistency of these patterns and determine whether the observed variations are isolated or more common.

Additional limitations include the small number of control specimens for vascular, neural, and histological comparisons; variation in breed, age, and body size; the absence of female specimens; and the limited use of fluoroscopy. Only one bilateral hernia case was examined for neural structures, preventing broader conclusions regarding neuropathic involvement. Finally, all specimens were obtained post-euthanasia, precluding dynamic functional assessments. The postmortem nature of the specimens prevents definitive differentiation between pathological changes and individual variability and that this ambiguity necessarily constrains the strength of the conclusions. Despite these limitations, the combined imaging and dissection approach provides valuable insights into the anatomical alterations associated with PHs in dogs. The contrast protocol used here is not applicable to living patients due to the high volume of iodine-based contrast required, which poses risks for older animals with renal disease.

The results of this study lay the groundwork for future research. Increasing sample size and including a wider variety of breeds could clarify whether the patterns identified here are consistent across the canine population or influenced by individual morphology. Advanced imaging modalities such as CT angiography, high-resolution MRI, or contrast-enhanced ultrasound could provide a more detailed in vivo characterization of vascular displacement or nerve entrapment. Longitudinal clinical studies may help determine how anatomical alterations evolve over time and whether they correlate with recurrence rates or surgical outcomes. Finally, the anatomical insights gained here may support refinement of surgical techniques, development of nerve-sparing approaches, and creation of educational resources to enhance training in PH repair.

## 5. Conclusions

This study provides a detailed description of the vascular, neural, and muscular alterations associated with perineal hernias in dogs by combining fluoroscopic imaging with anatomical dissection. Comparison of control material with unilateral and bilateral hernias cases showed that progressive degeneration of the pelvic diaphragm can markedly and unpredictably displace the dorsal and ventral PAs, the internal iliac and pudendal arterial systems, and the superficial perineal nerve. In advanced cases, particularly bilateral hernias, these changes were further compounded by organ migration. The close correspondence between fluoroscopic and anatomical findings highlights the value of imaging as a complementary tool for anticipating individual anatomical configurations. Overall, the results emphasize the importance of individualized anatomical assessment during surgical planning and contribute to a better understanding of the pathophysiological processes underlying perineal hernia, with potential implications for improving diagnosis, surgical outcomes, and long-term management.

## Figures and Tables

**Table 1:** Characteristics of animals included in the study.

Animal ID	Breed	Sex	Weight (kg)	Age (Years)
Control 1	Cocker Spaniel	♂	14	12
Control 2	Podenco	♂	10	9
Case 1	Mixed breed	♂	12	14
Case 2	Mixed breed	♂	12	11
Case 3	Poodle	♂	5.5	15

## Data Availability

The original contributions presented in this study are included in the article. Further inquiries can be directed to the corresponding author.

## Supplementary Materials

The following supporting information can be downloaded at: https://www.mdpi.com/article/10.3390/vetsci13040353/s1, Video S1: Fluoroscopy of Case 1 dog. Video S2: Fluoroscopy of control dog.

## Abbreviations

The following abbreviations are used in this manuscript:

AS	External anal sphincter muscle
Cc	Coccygeal muscle
cdg	Caudal gluteal artery
cfcn	Caudal femoral cutaneous nerve
CGA	Caudal gluteal artery
cdr	Caudal rectal artery
crg	Cranial gluteal artery
ct	Common trunk
DG	Deep gluteal muscle
dpn	Deep perineal nerve
dp	Dorsal perineal artery
H&E	hematoxylin and eosin
HS	Hernia sac
ii	Internal iliac artery
IIA	Internal iliac artery
il	Iliolumbar artery
ip	Internal pudendal artery
IPA	Internal pudendal artery
IT	Ischial tuberosity
lcd	Lateral caudal artery
NAV	Nomina Anatomica Veterinaria
IO	Internal obturator muscle
p	Prostatic artery
PA	Perineal artery
PAs	Perineal arteries
PB	Penile bulb
PeR	Penile retractor muscle
PH	Perineal hernia
SG	Superficial gluteal muscle
spn	Superficial perineal nerve
SW	Sacral wing
UB	Urinary bladder
ur	Urethral artery
vp	Ventral perineal artery
vp′	Dorsal scrotal branch of the ventral perineal artery
